# Spontaneous unscarred uterine rupture in a twin pregnancy complicated by adenomyosis: A case report

**DOI:** 10.1097/MD.0000000000024048

**Published:** 2021-01-22

**Authors:** Xuqing Li, Caihua Li, Meiguo Sun, Hongyan Li, Yunxia Cao, Zhaolian Wei

**Affiliations:** a Department of Obstetrics and Gynecology, the First Affiliated Hospital of Anhui Medical University; b NHC Key Laboratory of Study on Abnormal Gametes and Reproductive Tract (Anhui Medical University; c Key Laboratory of Population Health Across Life Cycle (Anhui Medical University), Ministry of Education of the People's Republic of China; d Anhui Province Key Laboratory of Reproductive Health and Genetics; e Biopreservation and Artificial Organs, Anhui Provincial Engineering Research Center, Anhui Medical University, Hefei, China.

**Keywords:** adenomyosis, spontaneous uterine rupture, twins

## Abstract

**Introduction:**

Uterine rupture during pregnancy is a serious obstetric complication accompanied by a high incidence of maternal morbidity and mortality, and the presence of uterine scars is the main risk factor. In the present case, uterine rupture occurred in an unscarred uterus in a nonlaboring primigravida woman with adenomyosis and twin pregnancy in the third trimester.

**Patient concerns:**

In this case, the patient suspected to have a history of endometriosis have got twin pregnancies following intracytoplasmic sperm injection, and complained of recurrent lower abdominal pain from 16 weeks to 29 weeks of gestation.

**Diagnosis:**

After exploratory laparotomy, the patient was diagnosed with uterine rupture and adenomyosis.

**Interventions:**

The patient was first administered expectant treatment such as antibiotics, tocolytics, and fluid replacement therapy. Symptoms then appeared repeatedly and worsened, followed by eventual peritoneal irritation, and exploratory laparotomy was performed.

**Outcomes:**

Two live female fetuses were extracted by cesarean section, and the uterine laceration was repaired. The mother recovered without any postoperative complications, and the babies were discharged after receiving one month of prematurity care without any postnatal complications.

**Conclusion:**

Adenomyosis and the conception of twins may lead to uterine rupture. For pregnant women with a history of adenomyosis with multiple gestations, close monitoring for signs of uterine rupture is necessary. Single-embryo transfer and multifetal pregnancy reduction should be recommended for infertile patients with adenomyosis.

## 1 Introduction

Uterine rupture is a catastrophic obstetric emergency associated with a high incidence of fetomaternal morbidity and mortality. The presence of a uterine scar is the main risk factor of uterine rupture. A paper published in 2015 reported incidences of ruptured uteruses in unscarred and scarred uteruses varying from 4.54 and 28.60 per 100,000 deliveries, respectively.^[1]^ Uterine rupture mostly occurs during the third trimester of pregnancy or during delivery in the presence of a uterine scar resulting from a previous cesarean delivery or myomectomy. It is extremely rare in the first or second trimester of pregnancy, and may vary in its presentation and course of events, which make the clinical diagnosis challenging. Advanced maternal age, multiparity, placenta increta, macrosomia, uterine malformation, shoulder dystocia, rare intrauterine manipulations such as internal podalic version, breech extraction, and medical termination of pregnancy are some other important contributing factors to this complication.^[2]^

Adenomyosis is characterized by heterotopic endometrial glands and stroma located in the myometrium presenting with menorrhagia, dysmenorrhea, and pelvic pressure symptoms. The ectopic endometrium tissue exhibits periodic hyperplasia and shedding under the influence of ovarian hormones. Adenomyosis, although a common gynecological condition at reproductive age, may rarely result in spontaneous rupture of an unscarred uterus during pregnancy.^[3]^ Twelve cases of rupture in a gravid uterus related to adenomyosis have been reported.^[4]^ Therefore, adenomyosis is considered a possible cause of uterine rupture.

In the present case, uterine rupture had occurred in an unscarred uterus in a nonlaboring primigravida woman in the third trimester without any intervention which is rarely encountered. Adenomyosis and twin pregnancy are possible risk factors. Our aim is to create awareness through this case report to avoid delays in diagnosis and timely management in such cases.

## 2 Case report

The patient was a 31-year-old female woman (gravid 1, para 0) with a history of primary infertility for 5 years due to male factor teratospermia. She was suspected to have endometriosis because of dysmenorrhea and a high cancer antigen 125 level, but no obvious endometriosis-like lesions were found on ultrasonography. Therefore, the couple underwent an intracytoplasmic sperm injection procedure at our center, resulting in conception of dichorionic diamniotic twins.

At 16 weeks of gestation, when she was 32 years old, she was first admitted to the gastroenterology department of our hospital complaining of recurrent intermittent lower abdominal pain and vomiting. She was discharged after her symptoms relieved following the treatment with intravenous ranitidine and fluids.

At 24 weeks of gestation, she was admitted again because of similar complaints that were relieved with symptomatic management; however, a hybrid echo-mass approximately 3.6 × 2.6 mm in size was found in the pelvic cavity on urgent ultrasonography.

At 28 weeks and 2 days of gestation, she was then readmitted with the same previous complaint of recurrent lower abdominal pain and vomiting. Urgent ultrasonography revealed 2 fetuses with regular heartbeats and an average amount of amniotic fluid. In addition, a hyperechoic solid (6.8 × 5.8 × 7.0 cm) was observed behind the cervix. Furthermore, she underwent magnetic resonance imaging (MRI), which revealed an irregular region of mixed T1 and T2 signals behind the cervix approximately 4.2 × 5.4 × 3.9 cm in size in the pelvic cavity, which was suspected to be a teratoma by an imaging physician based on previous experience and the normal appearances of the appendix, bladder, and kidneys. In addition, she was administered expectant treatment such as antibiotics and tocolytics to inhibit uterine contractions and drugs to promote fetal lung maturity. Lastly, her symptoms relieved again.

Five days after discharge, at 29 weeks and 6 days of gestation, she complained of a sudden onset of acute abdominal pain accompanied by vomiting and diarrhea. On general examination, her vital signs were stable, and generalized tenderness and rebounding were noted in the right lower quadrant. Ultrasonography revealed a fluid dark area approximately 58 × 62 mm in size with a floating light spot and abundant blood flow signal behind the cervix. The patient was counseled concerning the possibility of peritoneal irritation, and informed consent for abdominal exploration was obtained.

**Figure 1:**
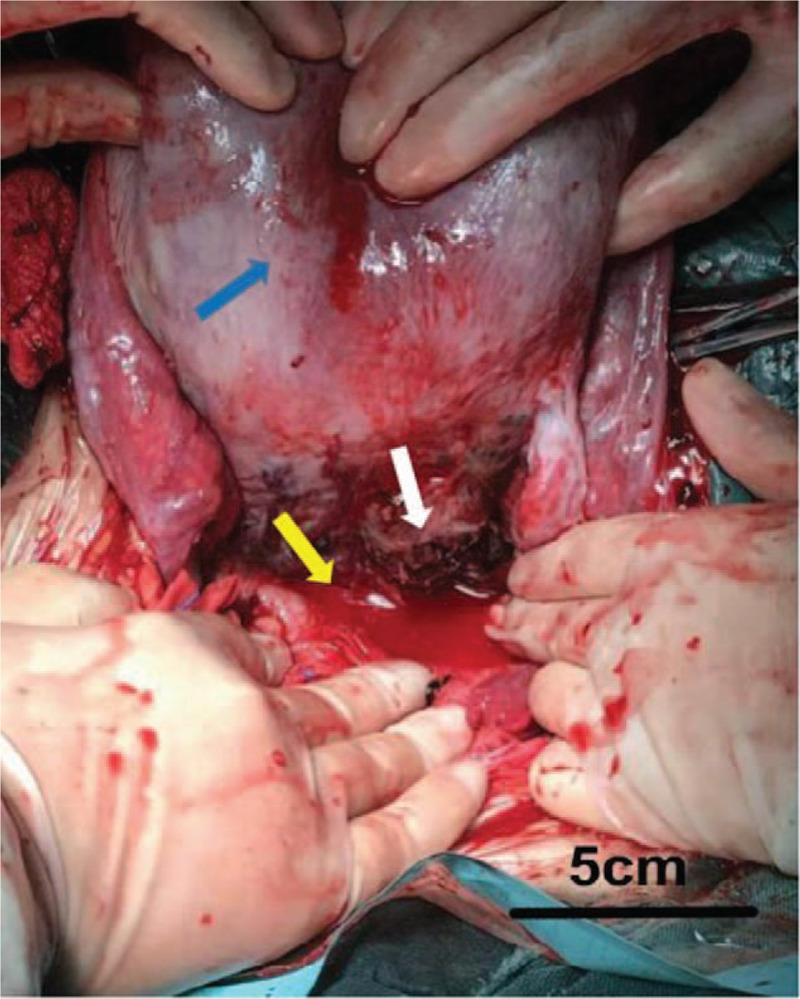
Intraoperative photograph of the uterus. Uterus was at 34th weeks gestation size (blue arrowhead). Myometrium and serosa are torn on the left posterior wall of the uterus. The laceration is 2 cm in length and does no penetrate into the uterine cavity (white arrowhead). There were approximately 500 ml dark red blood and about 50 g fresh clots were found in the paracolecular groove (yellow arrowhead). Bar: 5 centimeters.

Exploratory laparotomy was performed under general anesthesia. After entering the peritoneal cavity, approximately 500 mL of dark red blood and approximately 50 g of fresh clots were found in the paracolecular groove on both sides. Two 1440-g and 1310-g female fetuses were extracted by cesarean section, with Apgar scores of 3 and 5 at 1 minute, and 8 and 7 at 5 minutes, respectively. The size of the uterus indicated that it was at 34 weeks of gestation with no abnormalities in the anterior wall, fundus of uterus, bilateral tubes, and ovaries. The Douglas fossa and sacral ligaments were abnormally thickened. The 2 infants were then transferred to the neonatal intensive care unit. Further exploration of the peritoneal cavity revealed extensive purplish-blue nodules at the peritoneum covering the lower posterior wall of the uterus with prominent vascularity. The obstetrician suspected adenomyosis approximately 4 × 3 × 1 cm in size in the posterior wall of the uterus near the right sacral ligament (Fig. 1) because the nodular hyperplasia was indistinct from the surrounding uterine tissue, and a 2-cm laceration on the adenomyoma was surprisingly found. Moreover, no cysts were found during explorations of the abdomen or adnexa. According to the Revised American Society for Reproductive Medicine classification of endometriosis, the disease was then classified as stage II (8 points).^[5]^ The laceration was not bleeding significantly and was repaired using absorbable sutures. Histological examination of uterine tissue along the ruptured area revealed a focus of adenomyosis. The patient was transfused with 2 units of packed red blood cells and a full course of antibiotics after surgery. She recovered and was discharged without any postoperative complications on the fourth postoperative day. The babies were discharged after receiving 1 month of prematurity care without any postnatal complications.

## 3 Discussion

Uterine rupture is defined as separation of the entire thickness of the uterine wall, with extrusion of fetal parts and intra-amniotic contents into the peritoneal cavity.^[6]^ It can occur in the second stage of labor or spontaneously during pregnancy without any signs of labor. The prevalence rate of rupture of a pregnant uterus during any time of pregnancy is 0.05%.^[7]^ A history of uterine surgery, such as cesarean delivery and myomectomy, is the most common risk factor of uterine rupture.^[8]^ Other contributing factors such as mismanaged labor, misuse of oxytocic drugs such as the prostaglandin misoprostol, previous uterine perforation, macrosomic hydrocephalic fetus, excessive uterine pressure, instrumental delivery such as internal podalic version, trauma, multiple gestations, placenta percreta, grand multiparity, and uterine anomalies may precipitate uterine rupture.^[2]^

In our case report, there were 2 risk factors considered to be correlated with uterine rupture in a primigravida woman with no history of uterine scars as follows:

### 3.1 Presence of adenomyosis at the site of uterine rupture

Adenomyosis is a common disease among women in the childbearing period and is characterized by the presence of the endometrial gland and stroma in the myometrium. It is an established cause of menorrhagia, dysmenorrhea, and pelvic pressure symptoms.^[9]^ However, it is most often asymptomatic, being incidentally discovered on ultrasound or laparoscopy examination.

It is commonly believed that pregnancy has beneficial effects on adenomyosis by reducing the symptoms attributed to decidualization. However, it has been reported in some cases with severe complications due to adenomyosis such as spontaneous uterine rupture.

There are 2 patterns of response of the adenomyotic stroma to pregnancy-related hormones. One is superficial foci of adenomyosis, which are located within 2 low-power fields from the basal layer of the endometrium with non to minimal decidualization, while the other is deeper foci of adenomyosis, which exhibit prominent decidualization.^[3]^ Higher expression of progesterone receptors in the stromal elements of adenomyosis is likely associated with the resultant stromal decidualization and supports the theory of response of adenomyosis to progesterone in pregnancy.^[4]^ Furthermore, it is evident that abundant decidual transformation of stromal cells in adenomyosis leads to atrophy and necrosis of muscle cells.^[10]^ The necrosis of uterine muscles causes atony and life-threatening rupture through separation of muscle cells.^[11]^

In 1942, Haydon first suggested that adenomyosis of the pregnant uterus increases the risk of uterine rupture, uterine atony, and complications of labor.^[12]^ And atony of uterine muscle could be caused by decidual transformation in adenomyosal fields, leading to a decrease in myometrial contractions and prolonged postpartum bleeding, for which hysterectomy is performed.^[11]^ Azziz reviewed 11 cases of uterine rupture from the past 80 years, 7 of which were causally related to adenomyosis.^[3]^ Uccella et al^[13]^ reviewed the literature between 1952 and 2011 and found that 1 of 25 reported cases of prelabor spontaneous uterine rupture involved adenomyosis. Mueller et al reported a 30-year-old primigravida woman who experienced spontaneous uterine rupture at 18 weeks of gestation due to heavily decidualized adenomyosis.^[14]^ In the case reported by Nikolaou, a patient experienced spontaneous uterine rupture of an unscarred uterus caused by multiple foci of adenomyosis with a marked decidual reaction in the adenomyotic stroma.^[4]^ Recently, Indraccolo et al reported a 37-year-old woman who experienced a not-in-labor primitive uterine rupture caused by adenomyosis.^[15]^ Based on the literature, we feel that adenomyosis may cause uterine rupture.

Histological examination of the uterus and gross observation revealed adenomyosis in the present case. Consistent with previous reports, uterine rupture in the patient may have been associated with adenomyosis. Transmural adenomyotic foci with marked decidualization and subsequent splaying of the myometrial smooth muscle fibers were likely responsible for the weakness of the myometrium and eventually uterine rupture.

### 3.2 Diamniotic dichorionic twin pregnancy after in vitro fertilization and embryo transfer

Twin pregnancies are a common complication of assisted reproductive technology. The uterus of a twin pregnancy is markedly enlarged with an increase in uterine filling and pressure, which may have precipitated the mid-trimester uterine rupture in the present case.

Rapid development of polyhydramnios resulting in overdistension of the uterus has been reported as the only known risk factor for a lateral uterine rupture in a singleton pregnancy.^[16]^ There have been some cases of uterine rupture with twin pregnancies in women with a prior cesarean section, placenta percreta, and histories of myomectomy and adenomyomectomy.^[17–21]^ Therefore, we can hypothesize that the overdistension of the uterus due to the twin pregnancy on an unripened cervix in the presence of adenomyosis may have predisposed posterior uterine rupture in the present case.

### 3.3 Diagnosis and management of uterine rupture

Preoperative diagnosis is a challenge for spontaneous antenatal uterine rupture. The most common symptoms are sudden onset of severe abdominal pain, fetal distress, vaginal bleeding, maternal hypovolemic shock, uterine atony, fever, and gastrointestinal discomfort.^[22]^ Furthermore, there are various misleading factors that cause delays in diagnosis and management, such as silent uterine rupture with no specific signs and symptoms, unstable clinical presentation during pregnancy, and various symptoms according to the site of rupture. Therefore, only surgical intervention can provide a definitive diagnosis.

Ultrasound examination can be used to confirm hemoperitoneum, but its utility in the diagnosis of uterine rupture is limited. In addition, MRI has been shown to be useful in evaluations of uterine wall integrity.^[23]^ Before establishing a definitive diagnosis of uterine rupture, other possibilities should be excluded, such as acute appendicitis, perforation of the bowel, subclinical chorioamnionitis, placental abruption, and nephrolithiasis.

Management of uterine rupture during pregnancy differs according to conditions of the mother and fetus. Cesarean delivery with uterine repair or hysterectomy is the traditional approach for saving the lives of a mother and fetus.^[24]^ However, when the age of the fetus is younger than the viability age, management decisions are complex, such as the repair of uterine rupture with successful delay in delivery.^[25]^ Repair of uterine rupture helps to preserve the reproductive health of nullipara patients with a recurrence risk of uterine rupture of 4% to 19% at the subsequent pregnancy.^[26]^ Therefore, it has been recommended that women with previous uterine rupture should seek an early cesarean delivery after assurance of fetal lung maturity.^[27]^

## 4 Conclusions

In the present case, it can be assumed that adenomyosis and twins lead to uterine rupture. This case emphasizes the importance of the possibility of spontaneous uterine rupture in pregnant women with a history of adenomyosis with multiple gestations. Therefore, close monitoring of the signs of uterine rupture during pregnancy is necessary. Thus, single-embryo transfer and multifetal pregnancy reduction should be recommended for infertile patients with adenomyosis to reduce the risk of uterine rupture. Furthermore, close observation of uterine contractions and sonographic evaluations are needed for pregnant women with adenomyosis.

## Author contributions

XXXX.

## References

[1] Gibbins KJ Weber T Holmgren CM Maternal and fetal morbidity associated with uterine rupture of the unscarred uterus. Am J Obstet Gynecol 2015;213:382.e1–6. 26026917 10.1016/j.ajog.2015.05.048

[2] Vernekar M Rajib R Unscarred uterine rupture: a retrospective analysis. J Obstet Gynaecol India 2016;66: Suppl 1: 51–4. 27651577 10.1007/s13224-015-0769-7 PMC5016409

[3] Azziz R Adenomyosis in pregnancy. A review. J Reprod Med 1986;31:224–7. 3712359

[4] Nikolaou M Kourea HP Antonopoulos K Spontaneous uterine rupture in a primigravid woman in the early third trimester attributed to adenomyosis: a case report and review of the literature. J Obstet Gynaecol Res 2013;39:727–32. 23151226 10.1111/j.1447-0756.2012.02042.x

[5] Revised American Society for reproductive medicine classification of endometriosis: 1996. Fertil, Steril 1997;67:817–21. 9130884 10.1016/s0015-0282(97)81391-x

[6] Bucklin BA Vaginal birth after cesarean delivery. Anesthesiology 2003;99:1444–8. 14639160 10.1097/00000542-200312000-00029

[7] Hofmeyr GJ Say L Gulmezoglu AM WHO systematic review of maternal mortality and morbidity: the prevalence of uterine rupture. BJOG 2005;112:1221–8. 16101600 10.1111/j.1471-0528.2005.00725.x

[8] Walsh CA Baxi LV Rupture of the primigravid uterus: a review of the literature. Obstet Gynecol Surv 2007;62:327–34. quiz 353–324. 17425811 10.1097/01.ogx.0000261643.11301.56

[9] Ferrero S Anserini P Abbamonte LH Fertility after bowel resection for endometriosis. Fertil Steril 2009;92:41–6. 18684451 10.1016/j.fertnstert.2008.04.070

[10] Villa G Mabrouk M Guerrini M Uterine rupture in a primigravida with adenomyosis recently subjected to laparoscopic resection of rectovaginal endometriosis: case report. J Minim Invasive Gynecol 2008;15:360–1. 18439512 10.1016/j.jmig.2007.10.011

[11] Jovanovic B Petrovic A Petrovic B Decidual transformation in adenomyosis during pregnancy as an indication for hysterectomy. Med Pregl 2009;62:185–8. 19623852 10.2298/mpns0904185j

[12] Schindler AE Buhler K Mettler L Treatment of endometriosis with the GnRH agonist buserelin (Suprecur): a multicenter study. Geburtshilfe Frauenheilkd 1994;54:569–73. 8001754 10.1055/s-2007-1022341

[13] Uccella S Cromi A Bogani G Spontaneous prelabor uterine rupture in a primigravida: a case report and review of the literature. Am J Obstet Gynecol 2011;205:e6–8. 10.1016/j.ajog.2011.08.013 22035954

[14] Mueller MD Saile G Bruhwiler H Spontaneous uterine rupture in the 18th week of pregnancy in a primigravida patient with adenomyosis. Zentralbl Gynakol 1996;118:42–4. 8588451

[15] Indraccolo U Iannicco A Micucci G A novel case of an adenomyosis-related uterine rupture in pregnancy. Clin Exp Obstet Gynecol 2015;42:810–1. 26753492

[16] Oquendo Cortez M Beltran Montoya J Soriano Ortega K Spontaneous uterine rupture in a patient with polyhydramnios as only risk factor. Case report, literature review and institutional experience. Ginecol Obstet Mex 2008;76:217–20. 18798421

[17] Bhikha-Kori G Sueters M Middeldorp JM Uterine rupture at 21 weeks in twin pregnancy with TTTS and previous C-section. Case Rep Obstet Gynecol 2017;2017:2690675. 28840046 10.1155/2017/2690675 PMC5559912

[18] Farooq F Siraj R Raza S Spontaneous uterine rupture due to placenta percreta in a 17-week twin pregnancy. J Coll Physicians Surg Pak 2016;26:121–3. 28666503

[19] Tsankova M Nikolov A Bosev D Spontaneous uterine rupture in third trimester twin ivf pregnancy following myomectomy. Akush Ginekol (Sofiia) 2012;51:50–3. 23234036

[20] Wada S Kudo M Minakami H Spontaneous uterine rupture of a twin pregnancy after a laparoscopic adenomyomectomy: a case report. J Minim Invasive Gynecol 2006;13:166–8. 16527723 10.1016/j.jmig.2005.12.002

[21] Tutschek B Hecher K Somville T Twin-to-twin transfusion syndrome complicated by spontaneous mid-trimester uterine rupture. J Perinat Med 2004;32:95–7. 15008396 10.1515/JPM.2004.018

[22] Surico D Amadori R Vigone A Successful delivery after surgical repair of uterine rupture at 15 weeks of gestation: case report and brief review. Eur J Obstet Gynecol Reprod Biol 2016;204:5–8. 27459146 10.1016/j.ejogrb.2016.05.034

[23] Furey EA Bailey AA Pedrosa I Magnetic resonance imaging of acute abdominal and pelvic pain in pregnancy. Top Magn Reson Imaging 2014;23:225–42. 25099561 10.1097/RMR.0000000000000029

[24] Martin JN Jr Brewer DW Rush LV Jr Successful pregnancy outcome following mid-gestational uterine rupture and repair using Gore-Tex soft tissue patch. Obstet Gynecol 1990;75 (3 Pt 2):518–21. 2304727

[25] Hawkins L Robertson D Frecker H Spontaneous uterine rupture and surgical repair at 21 weeks gestation with progression to live birth: a case report. BMC Pregnancy Childbirth 2018;18:132. 29728141 10.1186/s12884-018-1761-x PMC5935985

[26] Ahmadi S Nouira M Bibi M Uterine rupture of the unscarred uterus. About 28 cases. Gynecol Obstet Fertil 2003;31:713–7. 14499715 10.1016/s1297-9589(03)00212-1

[27] Conturso R Redaelli L Pasini A Spontaneous uterine rupture with amniotic sac protrusion at 28 weeks subsequent to previous hysteroscopic metroplasty. Eur J Obstet Gynecol Reprod Biol 2003;107:98–100. 12593906 10.1016/s0301-2115(02)00242-7

